# Letter to the Editor: The causal effects of primary biliary cholangitis on irritable bowel syndrome: a Mendelian randomization study

**DOI:** 10.1097/JS9.0000000000002802

**Published:** 2025-07-02

**Authors:** Shuzheng Yuan, Mengying Bai, Hao Sun

**Affiliations:** a Department of Emergency, Shenzhen Longhua District Central Hospital, Shenzhen, Guangdong, China; b Shenzhen Traditional Chinese Medicine Hospital, Shenzhen, Guangdong, China; c Wolfson Institute for Biomedical Research, UCL Division of Medicine, University College London, London, UK

*Dear Editor,*

We read with great interest the recent article published in your journal investigating the potential causal relationship between primary biliary cholangitis (PBC) and irritable bowel syndrome (IBS) using a two-sample Mendelian randomization (MR) approach^[1]^. This study addresses a significant gap in the current understanding of the gut–liver axis by leveraging genetic data to explore the intersection between autoimmune liver disease and functional gastrointestinal disorders. The authors are to be commended for their rigorous methodological design, including the careful selection of instrumental variables, comprehensive sensitivity analyses, and robust pleiotropy assessments. While the study makes a valuable contribution, we would like to offer a few constructive suggestions that may further enhance the academic impact and clinical relevance of the work:

## Effect size and clinical significance

The reported causal association between PBC and IBS, while statistically significant (OR ≈ 1.01), appears to be of relatively small magnitude. This modest effect size may limit its immediate clinical implications. Additionally, the analysis relies on a single dataset for each phenotype. Considering the availability of multiple large-scale GWAS datasets (UK Biobank, FinnGen, GCST006304)^[2]^, we suggest that incorporating data from additional sources or validating the findings with real-world clinical datasets could improve the generalizability and clinical interpretability of the results.

## Phenotypic heterogeneity of IBS

IBS is a clinically heterogeneous condition, comprising subtypes such as diarrhea-predominant (IBS-D), constipation-predominant (IBS-C), and mixed-type (IBS-M)^[3]^. These subtypes may differ in terms of immune involvement and gut microbiota composition. Future studies could benefit from stratifying IBS cases by subtype, where data availability permits, to determine whether the causal effect of PBC is more pronounced in specific subgroups.

## Lack of empirical support for biological mechanisms

While the authors have provided a thoughtful discussion of the gut–liver axis, the proposed mechanisms remain largely theoretical. We recommend integrating functional annotation resources (GTEx, eQTL, mQTL databases) to investigate whether the identified SNPs influence genes involved in immune regulation, bile acid metabolism, or intestinal barrier integrity. In addition, transcriptomic comparisons between PBC and IBS could help reveal overlapping molecular signatures and provide biological validation for the shared genetic signals.

## Consideration of population and sex differences

PBC shows a strong female predominance, particularly in middle-aged and older women^[4]^, and sex-related differences may also influence IBS pathophysiology. Stratified analyses by sex, as permitted by datasets such as the UK Biobank, or a clearer discussion of the demographic composition of the current sample could help clarify the scope and limitations of the study’s findings.

In conclusion, this study offers important genetic evidence supporting a potential causal link between PBC and IBS. We sincerely appreciate the authors’ efforts and encourage future work that extends these findings through mechanistic validation, phenotype refinement, and clinical translation.

## Data Availability

Not applicable.
